# Supplementary material for: Not all Medicaid for pregnancy care is delivered equally
Source: PLoS One. 2024 Apr 3;19(4):e0299818. doi: 10.1371/journal.pone.0299818 (PMC10990183; doi:10.1371/journal.pone.0299818)
Supplement: S1 File — (DOCX) [file pone.0299818.s002.docx]

**Supporting Information**

Not all Medicaid for pregnancy care is delivered equally

Jonas J. Swartz, MD, MPH, Menolly Kaufman, PhD, MPH, Maria I. Rodriguez, MD, MPH

##

## Supporting information Table. Medicaid Enrollment and Utilization by Medicaid Type and Perinatal Period, Oregon and South Carolina 2014-2019

|  | **Oregon** | | **South Carolina** | |
| --- | --- | --- | --- | --- |
|  | Pregnancy  (n = 58,044) | Traditional  (n = 47,764) | Pregnancy  (n = 85,479) | Traditional  (n = 52,907) |
| **Months of Enrollment (Mean (SD))** |  |  |  |  |
| Preconception | 1.2 (1.0) | 1.4 (0.9) | 0.5 (0.9) | 1.5 (1.0) |
| Prenatal | 8.9 (1.9) | 9.1 (1.9) | 8.2 (3.0) | 9.5 (3.4) |
| Postpartum (up to one year after delivery) | 9.4 (2.9) | 10.0 (2.5) | 7.3 (4.6) | 10.4 (4.0) |
| Postpartum (first 60 days after delivery) | 2.0 (0.1) | 2.0 (0.1) | 2.0 (0.6) | 2.0 (0.6) |
| Postpartum (61-365 days after delivery) | 7.4 (2.8) | 8.0 (2.4) | 5.2 (4.3) | 8.3 (3.5) |
| **Visits (N (%))** |  |  |  |  |
| At least one pre-conception visit | 3,041 (5.2) | 3,530 (7.4) | 5,783 (6.8) | 15,717 (29.7) |
| At least seven prenatal visits | 45,691 (78.7) | 40,076 (83.9) | 54,479 (63.7) | 36,350 (68.7) |
| At least one visit (first 60 days after delivery) | 35,509 (61.2) | 31,019 (64.9) | 62,326 (72.9) | 38,192 (72.2) |
| At least one visit (61-365 days after delivery) | 38,764 (66.8) | 35,013 (73.3) | 34.775 (40.7) | 36,866 (69.7) |

Notes: SD = Standard Deviation
