# Supplementary material for: Not all Medicaid for pregnancy care is delivered equally
Source: PLoS One. 2024 Apr 3;19(4):e0299818. doi: 10.1371/journal.pone.0299818 (PMC10990183; doi:10.1371/journal.pone.0299818)
Supplement: Attachment — Submitted filename: Reviewer response_9.29.docx [file pone.0299818.s004.docx]

Dear PLOS editors

Thank you for the opportunity to revise this manuscript. We have included our response to reviewers and the editors below.

Journal Requirements:

When submitting your revision, we need you to address these additional requirements.

1. Please ensure that your manuscript meets PLOS ONE's style requirements, including those for file naming. The PLOS ONE style templates can be found at

[https://journals.plos.org/plosone/s/file?id=wjVg/PLOSOne_formatting_sample_main_body.pdf](https://urldefense.com/v3/__https:/journals.plos.org/plosone/s/file?id=wjVg*PLOSOne_formatting_sample_main_body.pdf__;Lw!!OToaGQ!v2044EOMRLJVDEYz_5pj_XWFyZ3THAbqrQZvycsYjjmCGYcjvdt2_CTtY3Oi-o3omqIW_rIXPTldeaRmwudB$) and

[https://journals.plos.org/plosone/s/file?id=ba62/PLOSOne_formatting_sample_title_authors_affiliations.pdf](https://urldefense.com/v3/__https:/journals.plos.org/plosone/s/file?id=ba62*PLOSOne_formatting_sample_title_authors_affiliations.pdf__;Lw!!OToaGQ!v2044EOMRLJVDEYz_5pj_XWFyZ3THAbqrQZvycsYjjmCGYcjvdt2_CTtY3Oi-o3omqIW_rIXPTldeQq1O3eE$).

*We made changes to fit the journal style.*

2. Thank you for stating in your Funding Statement:

“This work was conducted with the support of award 1R01MD013648-01 for MIR from the National Institute on Minority Health and Health Disparities ([https://www.nimhd.nih.gov/](https://urldefense.com/v3/__https:/www.nimhd.nih.gov/__;!!OToaGQ!v2044EOMRLJVDEYz_5pj_XWFyZ3THAbqrQZvycsYjjmCGYcjvdt2_CTtY3Oi-o3omqIW_rIXPTldeWMUVkvu$)) and grant K12HD103083 for JJS from the National Institute of Child Health and Human Development (NICHD) ([https://www.nichd.nih.gov/](https://urldefense.com/v3/__https:/www.nichd.nih.gov/__;!!OToaGQ!v2044EOMRLJVDEYz_5pj_XWFyZ3THAbqrQZvycsYjjmCGYcjvdt2_CTtY3Oi-o3omqIW_rIXPTldeYn2s_Wv$)) of the U.S. National Institutes of Health. The content is solely the responsibility of the authors and does not necessarily represent the official views of the National Institutes of Health.
The funders had no role in study design, data collection and analysis, decision to publish, or preparation of the manuscript.”

Please provide an amended statement that declares *all* the funding or sources of support (whether external or internal to your organization) received during this study, as detailed online in our guide for authors at [http://journals.plos.org/plosone/s/submit-now.](https://urldefense.com/v3/__http:/journals.plos.org/plosone/s/submit-now.__;!!OToaGQ!v2044EOMRLJVDEYz_5pj_XWFyZ3THAbqrQZvycsYjjmCGYcjvdt2_CTtY3Oi-o3omqIW_rIXPTldeQf_QcNg$)  Please also include the statement “There was no additional external funding received for this study.” in your updated Funding Statement.

Please include your amended Funding Statement within your cover letter. We will change the online submission form on your behalf.

*We have updated the funding statement on the title page and in the cover letter.*

3. Your ethics statement should only appear in the Methods section of your manuscript. If your ethics statement is written in any section besides the Methods, please move it to the Methods section and delete it from any other section. Please ensure that your ethics statement is included in your manuscript, as the ethics statement entered into the online submission form will not be published alongside your manuscript.

*We moved the ethics statement.*


[Note: HTML markup is below. Please do not edit.]

Reviewers' comments:

Reviewer's Responses to Questions

Comments to the Author

1. Is the manuscript technically sound, and do the data support the conclusions?

The manuscript must describe a technically sound piece of scientific research with data that supports the conclusions. Experiments must have been conducted rigorously, with appropriate controls, replication, and sample sizes. The conclusions must be drawn appropriately based on the data presented.

Reviewer #1: Partly

Reviewer #2: Yes

2. Has the statistical analysis been performed appropriately and rigorously?

Reviewer #1: I Don't Know

Reviewer #2: Yes

3. Have the authors made all data underlying the findings in their manuscript fully available?

The [PLOS Data policy](https://urldefense.com/v3/__http:/www.plosone.org/static/policies.action*sharing__;Iw!!OToaGQ!v2044EOMRLJVDEYz_5pj_XWFyZ3THAbqrQZvycsYjjmCGYcjvdt2_CTtY3Oi-o3omqIW_rIXPTldeTSBMK2n$) requires authors to make all data underlying the findings described in their manuscript fully available without restriction, with rare exception (please refer to the Data Availability Statement in the manuscript PDF file). The data should be provided as part of the manuscript or its supporting information, or deposited to a public repository. For example, in addition to summary statistics, the data points behind means, medians and variance measures should be available. If there are restrictions on publicly sharing data—e.g. participant privacy or use of data from a third party—those must be specified.

Reviewer #1: No

Reviewer #2: Yes

4. Is the manuscript presented in an intelligible fashion and written in standard English?

PLOS ONE does not copyedit accepted manuscripts, so the language in submitted articles must be clear, correct, and unambiguous. Any typographical or grammatical errors should be corrected at revision, so please note any specific errors here.

Reviewer #1: Yes

Reviewer #2: Yes

5. Review Comments to the Author

Please use the space provided to explain your answers to the questions above. You may also include additional comments for the author, including concerns about dual publication, research ethics, or publication ethics. (Please upload your review as an attachment if it exceeds 20,000 characters)

Reviewer #1: This paper examines rates of outpatient care before, during, and after pregnancy among those enrolled in Medicaid via the pregnancy pathway versus all other pathways. They find that those who were enrolled in Medicaid via the pregnancy pathway had lower rates of preconception, prenatal, and postpartum visits than those in traditional Medicaid using linked claims and birth record data from Oregon and South Carolina. The study uses unique data and addresses an important and understudied topic, but could benefit from additional clarification and detail.
Major:
1. This study frames the outcomes as “access to care” in the perinatal periods, but this is a secondary outcome to what is driving access to care in this context, which is eligibility for Medicaid. Those who are eligible for Medicaid only due to pregnancy are by definition not eligible before or after pregnancy based on their incomes, so a large portion of those in this group will fall out of observation. It is unclear how the study’s methodology deals with this. Does the denominator of the rates include those who fall out of coverage or exclude them? It is therefore unclear whether the study is comparing access to care within Medicaid enrollees or comparing those who maintain coverage to those who drop out and care can therefore not be observed.

*We agree with the reviewer that one limitation of this methodology and dataset is that individuals could have unobserved care utilization that is not funded by Medicaid and, therefore, does not appear in claims. The denominator includes individuals enrolled in Medicaid at the time of birth. One opportunity for future research might be comparing enrollment patterns stratified by categorical eligibility. We added the following to the limitations to reflect the reviewer’s feedback: Line 202-204.*

Claims data also does not capture care obtained but not financed by Medicaid, so individuals could have received care unobserved in this dataset outside the time of their Medicaid enrollment.

a. If the rates includes those who drop out of coverage, they are comparing utilization of services among those who they have data for to those who are lost to follow-up due to lack of eligibility for the program. This makes the results difficult to interpret since a lack of outpatient care prior to or after pregnancy does not mean that no outpatient care was received, it just wasn’t paid for by Medicaid and therefore is unobservable in the data. This is an important distinction to make clearly and up front. This renders the findings less relevant because we would expect that those who are not in Medicaid would not have any Medicaid-financed care.

*We did include those who drop out of coverage, and have added the reviewer’s requested clarification to the limitations. (Line 202-204) We would suggest that the findings remain relevant because our objective is to characterize how different Medicaid programs shape access to care. While people may access care, prior data suggest that insurance lapses are common among women using Medicaid for pregnancy and that the most common pattern is periods of uninsurance rather than switching to private insurance.(1) Individuals may get care that is not captured during periods of uninsurance but it remains an impediment to care.*

b. However, if the denominator restricts to those who are enrolled due to pregnancy but also maintain their coverage prenatally and postpartum, this would imply that there is something about being enrolled in the pregnancy pathway specifically that results in lower outpatient use (and the authors should spend more time unpacking and hypothesizing what this is). However, if the authors do condition on enrollment, then the timing of assessment of eligibility pathway becomes highly relevant. If someone is enrolled in the pregnancy pathway during pregnancy but enrolled as a low-income parent after pregnancy, which group are they classified in? Are people switching eligibility pathway to pregnancy when they become pregnant? At what time point during pregnancy was eligibility pathway assessed?

*We classify enrollment category based on enrollment at the time of delivery. Future research might be fruitful examining potential switching of eligibility pathways, as the reviewer suggests. However, that analysis is not included in this study. We suspect the primary driver of differential utilization is that pregnancy Medicaid recipients enroll later and disenroll earlier.*

2. Relatedly, those who remain in Medicaid before and after pregnancy may be systematically different from those who are only enrolled due to pregnancy. This is because income eligibility limits for pregnancy are higher than for other groups, and therefore those who maintain insurance beyond pregnancy are typically lower income which may be associated with health status and other factors.

*We agree with the reviewer that the populations are different between traditional and pregnancy Medicaid populations and that some of these differences may affect need for and use of care. We have added the following to the discussion: Line 199-202*

Some difference in care utilization may be attributable to differences in the inidividuals eligible for Traditional Medicaid versus Pregnancy Medicaid. Those with Traditional Medicaid have lower incomes and could have more chronic conditions that could require increased visits, for example.

3. Were outpatient prenatal visits measured using the birth record or the claims? Prenatal care in the birth record would capture prenatal care not covered via Medicaid which would be one way to get around the issue of lack of observation of non-Medicaid financed care. Otherwise you don’t actually know that those who are not in the Medicaid program preconception or postpartum aren’t getting outpatient care via other insurance.

*We used claims data for utilization outcomes. We have added acknowledgement of this to the limitations. (Line 202-204)*

Claims data also does not capture care obtained but not financed by Medicaid, so individuals could have received care unobserved in this dataset outside the time of their Medicaid enrollment.

4. Did the study restrict to the pregnant population or to those who had claims that indicated a delivery/labor?

*Analysis was restricted to those with live births. This is noted in the methods section: (Line 85)*

This retrospective cohort study compared outpatient visits using linked birth certificate and Medicaid claims from all Medicaid live births in two states (Oregon and South Carolina).

5. The study groups eligibility into pregnancy versus traditional, but there are many different reasons for Medicaid eligibility beyond pregnancy which have different implications for utilization of services (i.e., disability, low-income adults, parents, children, etc). How were these grouped? What reasons for eligibility are categorized as “traditional”?

*Traditional eligibility included, as suggested by the reviewer, multiple categories including those providing coverage for low-income, blind and disabled individuals. We grouped all of these programs, which were not specifically for pregnancy, as “traditional.” We intended to make the distinction that enrollment for these programs was not preconditioned on pregnancy. We include the following for clarification. (Line 94-97)*

Each state has multiple programs unrelated to pregnancy that offer categorical eligibility for services (e.g. adults with dependent children, blindness or disability), provided enrollees are below income thresholds. These multiple enrollment categories comprise the traditional Medicaid population in this study.

6. More specific language needed in first paragraph – what is the difference between full benefits and comprehensive coverage?

*Thank you for this clarification. We have updated the language to clarify that we intended to highlight the difference in duration of eligibility, not the services available in the two programs. (Line 76-78)*

Pregnancy Medicaid generally includes access to comprehensive coverage and lasts from pregnancy diagnosis through 60 days postpartum while traditional Medicaid is comprehensive coverage irrespective of pregnancy.

7. How are you defining the perinatal period? There are specific definitions of this term so would suggest avoiding use of this term and just defining how you measured this period.

*We are interested in the 12 months before and after a birth. Thank you for this request. We have updated the language. (Line 79-81)*

Our objective was to determine the association between type of Medicaid (traditional Medicaid and pregnancy Medicaid) on receipt of outpatient care during the 12 months before and after a birth.

8. Results section is brief and does not describe differences by subgroups or across the different time points. This section reports the average number of visits but the outcome was assessed as a rate.
*We expanded the results section to discuss the demographic characteristics of the study population (new Table 1). We also expanded our results section, as requested*, *and now includes* *both rates and absolute number of visits.*
Minor:
1. The specific numerical results in the discussion section would fit better in the results section.

*These are now reported in the results section though some are also noted in the discussion when they are relevant.*

2. In the abstract it says that pregnancy-related Medicaid eligibility ends 60 days postpartum. This is no longer the case in the majority of states due to postpartum Medicaid extensions.

*Thanks for this note. We discuss this at the end of the paper with updated data on adoption across the US. However, it was the case at the time of data collection. We changed the language to clarify. (Line 50)*

Pregnancy Medicaid ended 60 days postpartum during the study.

3. Medicaid is the most common public payer but commercial is largest payer of births in the U.S.

*We have made our language more precise.(Line 67)*

Medicaid pays for over 40% of obstetric care in the United States (US)(2)

Reviewer #2

I would like to thank the authors for giving me the opportunity to review this paper.
The paper titled "Not all Medicaid for pregnancy care is delivered equally" by Swartz et al investigates the association between Medicaid types (Pregnancy Medicaid and Traditional Medicaid) and outpatient care utilization during the perinatal period in Oregon and South Carolina from 2014 to 2019.

The paper addresses an essential topic concerning disparities in healthcare access for pregnant Medicaid beneficiaries and presents valuable insights. Strengths of this study are:

- Addresses an important and relevant topic related to healthcare disparities among pregnant Medicaid beneficiaries.
- Utilizes a retrospective cohort design, linked birth certificate, and Medicaid claims data, enhancing the study's validity and robustness.
- Presents clear and easy-to-understand tables, including unadjusted and adjusted means, allowing for comprehensive interpretation of outpatient care utilization during the perinatal period.
- Effectively emphasizes the significance of preconception and postpartum care in improving maternal and fetal health outcomes.
- Acknowledges the potential implications of lacking coverage during critical periods, such as missed opportunities for accessing healthcare and contraception.
- Provides valuable insights into outpatient care utilization for both Medicaid types (Pregnancy Medicaid and Traditional Medicaid) in two states (Oregon and South Carolina) from 2014 to 2019.
- Highlights the importance of considering additional adjustment variables to account for potential confounding factors that could influence the study's findings.
- Recommends the inclusion of data from more states with diverse demographics and healthcare systems to enhance the generalizability of the results.
- Acknowledges the policy changes related to extended postpartum Medicaid coverage and suggests the need for further research in this area.

The paper's findings can contribute to policy discussions and potential positive changes in maternal healthcare access and outcomes for Medicaid beneficiaries.
In summary, this is a well-written and concise paper and I would recommend it for publication with some minor changes as follows:

1. Please provide a concise conclusion section to this paper which can help the reader to gain the main points of the study in a single glance.

*We added a conclusion.*

2. Line 104 mentions “ANCOVA…”. Do you mean analysis of variances (ANOVA)? Please clarify this to the reader.

*Thank you for this question. We used analysis of covariance so that we could compare differences in means while controlling for covariates/confounders. We have updated the language in the document.(Line 109-110)*

We assessed for differences in utilization in the preconception, prenatal and postpartum periods using analysis of covariance (ANCOVA) allowing us to adjust for maternal age, parity, rurality, history of cesarean delivery, hypertensive disease of pregnancy and diabetes during pregnancy.

1.	Daw JR, Hatfield LA, Swartz K, Sommers BD. Women In The United States Experience High Rates Of Coverage ‘Churn’ In Months Before And After Childbirth. Health Affairs. 2017 Apr 1;36(4):598–606.

2.	Medicaid and CHIP Payment and Access Commission. Medicaid’s Role in Financing Maternity Care [Internet]. MACPAC; 2020 [cited 2022 May 23]. Available from: https://www.macpac.gov/wp-content/uploads/2020/01/Medicaid%E2%80%99s-Role-in-Financing-Maternity-Care.pdf
