# Supplementary material for: Not all Medicaid for pregnancy care is delivered equally
Source: PLoS One. 2024 Apr 3;19(4):e0299818. doi: 10.1371/journal.pone.0299818 (PMC10990183; doi:10.1371/journal.pone.0299818)
Supplement: Attachment — Submitted filename: Response to Reviewers 2nd revision.docx [file pone.0299818.s005.docx]

**Response to reviewers – 12.8.23**

Reviewer #1: I thank the reviewers for revisions. I think some more could be done to strengthen the relevance and impact of the study conclusions:
(1) It is unclear to me why the authors are not using the prenatal utilization data from the birth record since they could observe non-Medicaid financed prenatal care. It can be challenging to measure prenatal care in claims due to bundled maternity billing so the birth record seems like an important and overlooked advantage. Instead of adding a limitation, they should address this using their data.

*Thank you for your thoughtful review. While we agree that there are limitations to claims data for identification of prenatal visits, the advantage is that we were able to report the timing of visits during a pregnancy. In contrast, birth certificate data would only report the total number of visits. So, while this might improve capture of prenatal visits that were not identified because of bundled billing, we would be unable to determine when visits occurred. Additionally, use of claims allows us to use consistent methodology in identification of preconception and postpartum visits which would not be included in birth certificate data.*

*We added an explanation to the methods (Line 100-102)*

We used Medicaid claims rather than birth certificate data to identify prenatal visits because birth certificates report only the total number of visits, not the timing of visits during the pregnancy.

*And to the limitations Line 203-204*

Additionally, because maternity care is often bundled in global billing for delivery, we may have under-identified prenatal visits.

(2) I think the results would be more interpretable if they also reported the percent of the cohort with a preconception visit, adequate prenatal care, and postpartum visit since these will be less affected by outliers. The number of visits themselves seem less relevant since in the preconception and postpartum periods clinical recommendations are for one visit for most people.

*We agree with the reviewer that examining these variables in a binary fashion to indicate whether care did or did not meet recommendations adds to the strength of our results. We conducted this analysis as a sensitivity analysis included in a supplemental table and discuss it in the results section. (Line 135-140)*

Results were similar in a sensitivity analysis conducted with dichotomized indicators of preconception, prenatal and postpartum care demonstrating a higher percentage of patients with traditional Medicaid had at least one-preconception visit than those with pregnancy Medicaid in both states. (Supplemental Table 1) A higher proportion of traditional Medicaid recipients also had at least seven prenatal visits than those with pregnancy Medicaid in both states.

(3) It says emergency Medicaid recipients were excluded but they are in Table 1. Also why exclude them when the goal is to compare across eligibility categories? They are an important and understudied group, so I don’t see the rationale in a descriptive study.

*We agree that this is an important and understudied population. We include them in Table 1 as an important component of the birthing population. We decided not to include them in other portions of the analysis as it was harder to make apples-to-apples comparisons with such a different population. Access to care for this population is largely determined by a different policy mechanism, state adoption of the CHIP Unborn Child provision allowing coverage of prenatal care. This has been studied extensively, including in these two states (Oregon provides prenatal care coverage, South Carolina does not), with greater focus on key metrics of access to care and health outcomes. Additionally, Emergency Medicaid recipients often rely on non-Medicaid-funded care for prenatal visits, for example, which would not have been captured in this dataset.*

(4) Since all the outcomes are dependent on enrollment and the authors have access to enrollment data, I think it is critical that they report on mean months of enrollment during each of the time periods to contextualize their findings. If they wanted to go further, they could estimate models where they control for enrollment length to see if that is the primary explanatory variable of the differences in outcomes across eligibility groups.

*We agree with the reviewer that enrollment and utilization are correlated. We report the mean number of months of enrollment in the preconception, prenatal and postpartum periods in Supplemental Table 1. We did test our models controlling for enrollment and when we did so, our models produced errors due to collinearity between enrollment and utilization. When considering the relative importance of enrollment versus utilization, we opt to emphasize utilization as enrollment seems like an intermediate outcome compared to measured utilization of care. In Methods lines 104-105.*

We also measured the number of months of enrollment at each perinatal time point.

*In results lines 154-158*

In Oregon, pregnancy and traditional Medicaid recipients had similar mean months of enrollment in the preconception (pregnancy 1.2 months vs traditional 1.5 months), prenatal (pregnancy 8.9 months vs traditional 9.1 months) and 61 day to 365 day postpartum periods (pregnancy 7.4 months vs traditional 8.0 months). In South Carolina, pregnancy Medicaid recipients had fewer mean months of enrollment in the preconception (pregnancy 0.5 months vs 1.5 months), prenatal (pregnancy 7.3 months vs traditional 10.4 months) and 61 day to 365 day postpartum periods (pregnancy 5.2 months vs traditional 8.0 months). (Supplemental Table 1)

(5) I think that the main policy takeaway of this study is missing -- it needs to be made much more clear in the discussion and abstract that these enrollment patterns are driven by eligibility income limits for pregnancy vs other groups which create discontinuities in insurance before versus during pregnancy. I think more discussion of how these limits vary across states and potential policy recommendations to smooth coverage in light of the findings would greatly strengthen the paper.

We expanded the discussion and conclusion including the points suggested by the reviewer, lines 205-228. We hope this brings this work better into the context of what is known on the topic and highlights how changes, especially those in the American Recovery Plan, may affect care.

(6) I also think it is worth noting the fact that despite the fact that people are pregnant and in Medicaid, they are not enrolled in the pregnancy pathway when in theory, they should be. What are the possible implications of this for policy and for researchers who may rely on these categories to define pregnant enrollees?

*We have added an explanation for why it may be beneficial for Medicaid beneficiaries to be enrolled in traditional as opposed to pregnancy Medicaid. Regarding identification of pregnancies, the authors are not aware of research using only the pregnancy Medicaid category for identification of pregnancies. Rather, most research we are aware of uses an algorithm similar to that utilized in this paper starting with diagnosis, procedure and DRG codes. Lines 212-215.*

Traditional Medicaid may be a favorable program for pregnant Medicaid beneficiaries because they are not at risk of losing eligibility after pregnancy. Additionally, some states limit the scope of covered services under pregnancy Medicaid as compared to traditional Medicaid plans.(11)

(7) To align with typical language used by Medicaid programs, I would refer to it as “eligibility category” not enrollment category.

*We have updated throughout when discussing eligibility categories.*
