# Supplementary material for: Not all Medicaid for pregnancy care is delivered equally
Source: PLoS One. 2024 Apr 3;19(4):e0299818. doi: 10.1371/journal.pone.0299818 (PMC10990183; doi:10.1371/journal.pone.0299818)
Supplement: Attachment — Submitted filename: Reviewer response 3.docx [file pone.0299818.s006.docx]

Dear editors of PLOS One,

Thank you for the opportunity to revise “Not all Medicaid for pregnancy care is delivered equally” for a third time. We have responded below to the reviewer’s feedback below.

Sincerely

Jonas Swartz, MD, MPH

Reviewer #1: This is making me write 100 characters in order to be able to move past this screen so I am writing in this box

Reviewer #3: 1. Abstract, reporting as 4.5 fold decrease implies a 22% relative risk of preconception visits for Pregnancy Medicaid vs traditional Medicaid. I think this is a more standard way of presenting the findings and it makes it easier for me to understand the magnitudes. Line 135-137 reports a 4.5:1 ratio of visits for TM beneficiaries --- this is easier to interpret as well.

*We appreciate the reviewers feedback that our reporting was unclear. As this is a continuous measure, we opted to use what the reviewer suggested is easier to interpret language from the body of the paper (modified per reviewer recommendation in later comments.*

*“Discussion: In South Carolina, those using traditional Medicaid had 450% more preconception visits and 70% more postpartum visits compared with pregnancy Medicaid. In Oregon, those using traditional Medicaid had 200% more preconception visits and 29% more postpartum visits than individuals using pregnancy Medicaid.”(Line 59-63)*

2. Traditional Medicaid in South Carolina is a very different category than traditional Medicaid in Oregon, assuming that traditional Medicaid includes ACA Medicaid expansion to 138% FPL. Please be clear here.

*We stipulate specific eligibility thresholds in the Methods, line 102-104. Further discussed on lines 209-216.*

*In Oregon, adults earning up to 138% of the Federal Poverty Level (FPL) may enroll in traditional Medicaid, while in South Carolina, they may earn up to 67% of FPL.*

3. I am having a hard time conceptualizing the rate as individuals with Pregnancy Medicaid only during this time period are only eligible for 60 days post-partum --- is there a differential rate in visits during this time period by Medicaid eligibility type or is most of the difference in visit rate after Day 60. Anderson et al PMID 35508289 has a good discussion on postpartum eligibility churn in North Carolina. How often are members switching categories of eligibility during the study period?

*We acknowledge that one limitation of this analysis is that care may occur with payors other than Medicaid and not be captured in claims data. It is possible that those with pregnancy Medicaid who lose coverage after 60 days subsequently seek care elsewhere. This analysis does stratify by timing of postpartum care visits and we report, in Table 2, care up to 60 days and care from 61-365 days. We hope this addresses the reviewer’s first concern. Regarding churn, thank you for this excellent citation. We added this concept and reference to the discussion. (Line 232-233) The reviewer’s final question about members switching categories of eligibility during the study period is good question that is out of the scope of this analysis but would be an interesting question for a future study.*

*“Data from North Carolina indicate a larger proportion of those with pregnancy Medicaid were at risk of losing coverage by four months postpartum.(11)”*

4. What is the average week of initial pre-natal visit by category? I am curious if the results are being driven by both right and left hand censoring of the Pregnancy Medicaid population?

*The week of initiation of prenatal care by category would be an interesting question for future study. The reviewer is correct that the Pregnancy Medicaid population is less likely to be enrolled and thus less likely to access care before enrollment and after 60 days postpartum. We hoped with this manuscript to illustrate what that looked like from a utilization standpoint (number of visits). (Lines 212-214)*

*“We also did not analyze timing of initiation of prenatal care, though that would be an opportunity for future research.*”

Minor comment:
a) Lines 75-77 “majority of states” is that accurate, or is it better to say “all states…” as looking at the Kaiser Family Foundation data, all states have higher Pregnancy income thresholds than other categories of eligibility thresholds.

*South Dakota has the same threshold for traditional Medicaid and pregnancy. We have made the language more specific as requested. (Lines 84-86)*

*“States have multiple categories of Medicaid programs enrolling pregnant people. Recognizing the importance of obstetrical care, all states but South Dakota cover pregnant people through pregnancy Medicaid at higher income thresholds than non-pregnant adults.”*

b) Lines 163-165 --- is it better expressed to say “traditional Medicaid beneficiaries had an average of 29% more visits than pregnancy Medicaid beneficiaries” as the current sentence seems to imply a 29% increase in the probability of at least one visit by TM but the supporting data indicates a count difference. Same Lines 165-167 for South Carolina

*We adjusted the language in the discussion and abstract as suggested by the reviewer. (line 180-185)*

*Care access postpartum was also different depending on Medicaid eligibility, especially from 61-365 days postpartum. In Oregon those with traditional Medicaid had 29% more 61-365 days postpartum as those with pregnancy Medicaid (mean 8.3 visits (SE 0.09) vs 6.3 visits (SE 0.08)). In South Carolina, those with traditional Medicaid had 70% more visits 61-365 days postpartum as those with pregnancy Medicaid (mean 4.8 visits (SE 0.04) vs mean 2.3 visits (SE 0.03)).*

c) Figure 1 is hard to interpret as it seems that each panel resets to zero multiple times for each bar and the color distinctions are not clear and obvious. Perhaps a stacked bar chart might work better for each state?

*We appreciate this feedback about the interpretability of this data visualization. We prefer to keep this heatmap format. We have updated the figure legend to provide a more detailed explanation.*

***“Legend****: Outpatient visits among two Medicaid enrollment categories in Oregon (panel a) and South Carolina (panel b). Dashed lines demarcate the preconception period, delivery and 60 days after delivery as, during the study period, pregnancy Medicaid coverage ended at this time postpartum. Each vertical rectangle in the panel represents average number of outpatient visits per person per month. Darker color indicates a higher number of outpatient visits during that month.”*
