# Supplementary material for: Not all Medicaid for pregnancy care is delivered equally
Source: PLoS One. 2024 Apr 3;19(4):e0299818. doi: 10.1371/journal.pone.0299818 (PMC10990183; doi:10.1371/journal.pone.0299818)
Supplement: Attachment — Submitted filename: Reviewer comments-PONE-D-23-04247.docx [file pone.0299818.s003.docx]

**I would like to thank the authors for giving me the opportunity to review this paper.
The paper titled "Not all Medicaid for pregnancy care is delivered equally" by Swartz et al investigates the association between Medicaid types (Pregnancy Medicaid and Traditional Medicaid) and outpatient care utilization during the perinatal period in Oregon and South Carolina from 2014 to 2019**.

The paper addresses an essential topic concerning disparities in healthcare access for pregnant Medicaid beneficiaries and presents valuable insights. Strengths of this study are:

- Addresses an important and relevant topic related to healthcare disparities among pregnant Medicaid beneficiaries.
- Utilizes a retrospective cohort design, linked birth certificate, and Medicaid claims data, enhancing the study's validity and robustness.
- Presents clear and easy-to-understand tables, including unadjusted and adjusted means, allowing for comprehensive interpretation of outpatient care utilization during the perinatal period.
- Effectively emphasizes the significance of preconception and postpartum care in improving maternal and fetal health outcomes.
- Acknowledges the potential implications of lacking coverage during critical periods, such as missed opportunities for accessing healthcare and contraception.
- Provides valuable insights into outpatient care utilization for both Medicaid types (Pregnancy Medicaid and Traditional Medicaid) in two states (Oregon and South Carolina) from 2014 to 2019.
- Highlights the importance of considering additional adjustment variables to account for potential confounding factors that could influence the study's findings.
- Recommends the inclusion of data from more states with diverse demographics and healthcare systems to enhance the generalizability of the results.
- Acknowledges the policy changes related to extended postpartum Medicaid coverage and suggests the need for further research in this area.

The paper's findings can contribute to policy discussions and potential positive changes in maternal healthcare access and outcomes for Medicaid beneficiaries.
In summary, this is a well-written and concise paper and I would recommend it for publication with some minor changes as follows:

**1. Please provide a concise conclusion section to this paper which can help the reader to gain the main points of the study in a single glance.**

**2. Line 104 mentions “ANCOVA…”. Do you mean analysis of variances (ANOVA)? Please clarify this to the reader.**
